# A Vision-Driven Collaborative Robotic Grasping System Tele-Operated by Surface Electromyography

**DOI:** 10.3390/s18072366

**Published:** 2018-07-20

**Authors:** Andrés Úbeda, Brayan S. Zapata-Impata, Santiago T. Puente, Pablo Gil, Francisco Candelas, Fernando Torres

**Affiliations:** 1 Department of Physics, System Engineering and Signal Theory, University of Alicante, 03690 Alicante, Spain; andres.ubeda@ua.es (A.Ú.); brayan.impata@ua.es (B.S.Z.-I.); santiago.puente@ua.es (S.T.P.); pablo.gil@ua.es (P.G.); Francisco.Candelas@ua.es (F.C.); 2 Computer Science Research Institute, University of Alicante, 03690 Alicante, Spain

**Keywords:** surface electromyography, computer vision, grasping, assistive robotics

## Abstract

This paper presents a system that combines computer vision and surface electromyography techniques to perform grasping tasks with a robotic hand. In order to achieve a reliable grasping action, the vision-driven system is used to compute pre-grasping poses of the robotic system based on the analysis of tridimensional object features. Then, the human operator can correct the pre-grasping pose of the robot using surface electromyographic signals from the forearm during wrist flexion and extension. Weak wrist flexions and extensions allow a fine adjustment of the robotic system to grasp the object and finally, when the operator considers that the grasping position is optimal, a strong flexion is performed to initiate the grasping of the object. The system has been tested with several subjects to check its performance showing a grasping accuracy of around 95% of the attempted grasps which increases in more than a 13% the grasping accuracy of previous experiments in which electromyographic control was not implemented.

## 1. Introduction

Nowadays, robots can perform a variety of tasks to help human operators in their work [1]. The use of robots to collaborate with people with disabilities in industrial environments is a growing sector. For instance, several studies analyse the execution of manufacturing tasks by disabled people [2,3]. In this line, robotic assistive technologies have been successfully introduced following two different approaches. They are used to assist humans who have motor disabilities to perform daily activities. Typical examples are prosthetics devices and exoskeletons for motor substitution, or smart homes where household tasks are performed and controlled by automatic systems. These technologies also provide novel rehabilitation therapies to recover motor function and reduce further complications. Essentially, assistive technologies seek to improve the well-being of humans with disabilities [4].

The inclusion of assistive robotics in industrial applications contributes to the improvement of occupational health of human operators. Tele-operation systems increase the degree of assistance in dangerous manipulation tasks. Their goal is to make a system capable of mimicking and scaling the movements of a human operator in the control of a manipulator avoiding the risks of handling dangerous products or carrying out dangerous actions. Before including assistive technologies in industrial tasks, several teleoperation aspects must be considered. One of them is the feedback to the user, therefore the use of haptic interfaces [5] is critical to obtain a more natural feeling of the robot operation. Another important aspect is the additional assistance given to the user in the performance of the assigned task; focused, for instance, on the possibility of providing an amputee with the capability of performing bimanual tasks [6]. The need of interacting with the environment requires of vision systems to recognise the working place and provide a proper manipulation of the products [7].

A good option to achieve a proper tele-operated robotic manipulation is to implement solutions based on techniques that provide reliable control signals from the human operator. Surface electromyography (sEMG) allows a system to record the electrical activity of muscle contractions in a non-invasive way [8]. The use of this information to control external devices is called myocontrol. Myocontrol techniques have been usually developed to obtain a reliable actuation of assistive devices in the field of prosthetics. This actuation ranges from simple binary control commands to complex multidimensional control [9,10].

Complex techniques have been applied to multi-finger prosthetic devices and robotic hands. However, myocontrol is generally limited to a few hand grips and still unreliable in realistic environments [11]. To avoid these limitations, several approaches have been recently proposed. One option is to provide a proper sensory feedback to the subject to close the control loop [12,13]. However, this option is still limited to the low accuracy in the classification of complex biomechanical tasks. Another alternative is the introduction of multimodal control of the robotic actuation which may provide a good solution to the unreliability of multidimensional control. In this case, another control method, such as gaze-tracking or electrooculography, is combined with myocontrol to increase reliability and speed [14,15]. Its main disadvantage is the increased workload on the user as both interaction methods must be controlled simultaneously.

To solve the problems arisen from the previously described solutions, we propose the use of a shared control of the end effector of the robot arm. To achieve this, complex positioning and grasping tasks are performed by an alternative system and sEMG processing provides high-level commands. In this case, myocontrol will be combined with a vision-based grasping system.

Grasping is one of the most significant tasks which is performed by humans in everyday manipulation processes. In recent works, robots have been provided with the ability to grasp objects [16,17]. It is often possible to see robots autonomously grasping objects in many industrial applications in which the environment is not dynamic and where both geometry and pose of objects are known. Therefore, the proper pose of the robotic hand or gripper to grasp the object is computed only once. This process is repeated whenever it is needed. More recently, robots are beginning to be self-sufficient and they are reaching a great level of autonomy to work without human intervention in unstructured scenarios or with dynamics in which the kind of objects or their poses are unknown, for example in industrial applications as in [18] and in storage and logistic applications [19].

Many grasp methods have been made possible by the advances in visual perception techniques of the environment, both 2D [20] and 3D [21]. In general, both techniques combine computer vision algorithms and traditional machine learning, the first for the extraction of object features of the scene and the second for the recognition of the objects by comparison and classification of extracted features with features from a dataset of known objects. Thereby, visual perception has allowed robots to have the ability of grasping in a similar way to humans, though under certain conditions, making use of object recognition algorithms [22,23,24] and pose estimation algorithms [25,26]. Recently, a significant number of new approaches have been proposed to localize robotic grasp configurations directly from sensor data without estimating object pose using training databases of real objects [27] or synthetic objects (CAD models) as in [28].

However, currently it is still not possible to compare the ability of robots and humans to grasp objects in a generic way, for each and every situation. The main drawback of applying visual perception techniques to accomplish a completely autonomous grasping is the great variability of the kind of objects (geometric shape, pose and visual appearance such as color or texture) that can be present in an environment. This demands a large datasets of training data to implement a robust algorithm to avoid ambiguity in both recognition and location processes of the objects in the scene. The proposed system may solve both the more relevant issues of grasping and the complexity of multidimensional myoelectric control, by combining the visual-driven system with simple electromyographic analysis, based on ON/OFF sEMG commands.

## 2. System Architecture

### 2.1. Vision-Guided Robotic Grasping System

**Figure 1:**
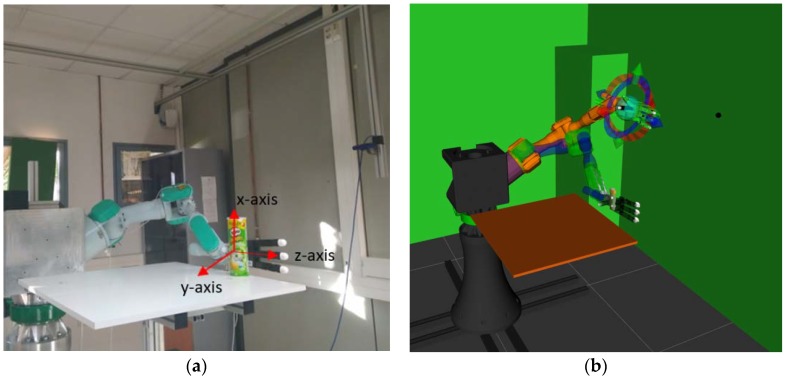
Pre-grasping pose of the robotic system computed by the vision algorithm. (**a**) Real robotic system in which the grasps are executed. (**b**) Simulation system where the movement is planned and the robotic hand pose is evaluated.

The system architecture is composed of a PA-10 industrial robot arm (Mitsubishi, Tokyo, Japan). This robot has seven degrees of freedom (DoF). The robot arm is controlled as a slave in a client-server software architecture managed from a Robot Operating System (ROS) framework. The PA-10 is connected to a server module installed on a computer acting as the PA-10 controller, and both elements are communicated via the Attached Resource Computer NETwork (ARCNET) protocol. The robot is always waiting for commands generated from the orders given by the computer vision algorithm running in the slave module. This module is also responsible for the planning and simulation of trajectories computed from the information obtained from the vision algorithm and from the data supplied by the sEMG system. In addition, the robot arm has an Allegro hand (Wonik Robotics, Seoul, Korea) attached to its end effector with a payload of 5 kg. It is a low cost and highly adaptive multi-finger robotic hand composed of 4 fingers and 16 independent torque-controlled joints, 4 for each finger. The Allegro hand is connected to the slave module via the Controller Area Network (CAN) protocol. The implementation of the system, with its different components, can be seen in Figure 1.

Additionally, the architecture of the system includes a RealSense Camera SR300 (Intel, Santa Clara, CA, USA). It is a depth-sensing camera that uses coded-light methodology for close-range depth perception. With this sensor, the system can acquire 30 colour frames per second with 1080 p resolution. SR300 is able to capture depth in a scenario from a distance between 0.2 m and 1.5 m. It is ideal to obtain shapes of real-world objects using point clouds.

### 2.2. Electromyography -Based Movement Control System for Robotic Grasping

After positioning the robot hand in front of the object, subjects perform a fine control of the grasping action by reorienting the end effector left or right and then provide the control output for the final approach to the object and subsequent robot hand closing. To obtain these control outputs surface electromyography has been recorded from the forearm during the performance of wrist flexion and extension.

**Figure 2:**
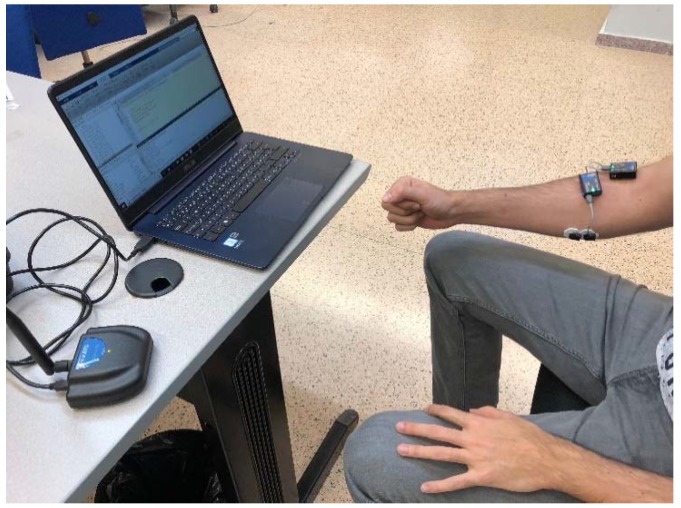
Surface electromyography (sEMG) system acquiring data from a subject.

**Figure 3:**
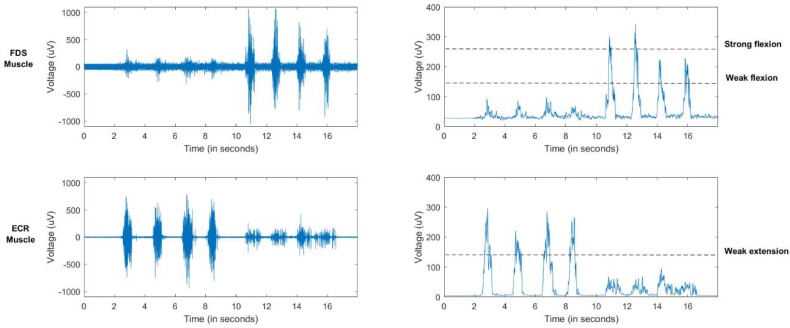
EMG raw signal for several flexion/extension wrist movements (**left**). Processed EMG signal and estimative thresholds (**right**).

To record surface electromyography (sEMG) signals a Mini DTS 4-channel EMG wireless system (Noraxon, Scottsdale, Arizona, USA) has been used (Figure 2). Two sEMG bipolar channels have been located over the *flexor digitorum superficialis* (FDS) and the *extensor carpi radialis longus* (ECR) of the forearm. Signals have been acquired with a sample frequency of 1500 Hz, then low-pass filtered below 500 Hz, full-wave rectified and, finally, smoothed with a mean filter of 50 ms (Figure 3).

Three different states have been classified from the filtered sEMG signal corresponding to a weak wrist flexion, a weak wrist extension and a strong wrist flexion. To classify these states, two thresholds have been defined to identify weak contractions (flexion on the FDS and extension on the ECR). Additionally, a higher threshold has been defined for strong contractions of the FDS (Figure 3). A ROS message is sent with the decoded output commands to the robotic system. This classification is performed every 0.5 s.

Weak flexion and extension is used to adjust the end effector in the z-axis (direction of the hand) with an initial step of 5 cm. These corrections can be performed through several control commands. When the robot end effector changes direction, the initial step is reduced to a 50%, which allows a fine adjustment of the position of the robot end effector avoiding a loop between end locations. Finally, when the operator thinks that the robot hand is properly positioned a strong flexion is used to perform the final approach to the object and the subsequent grip action.

## 3. Proposed Method for Grasping

**Figure 4:**
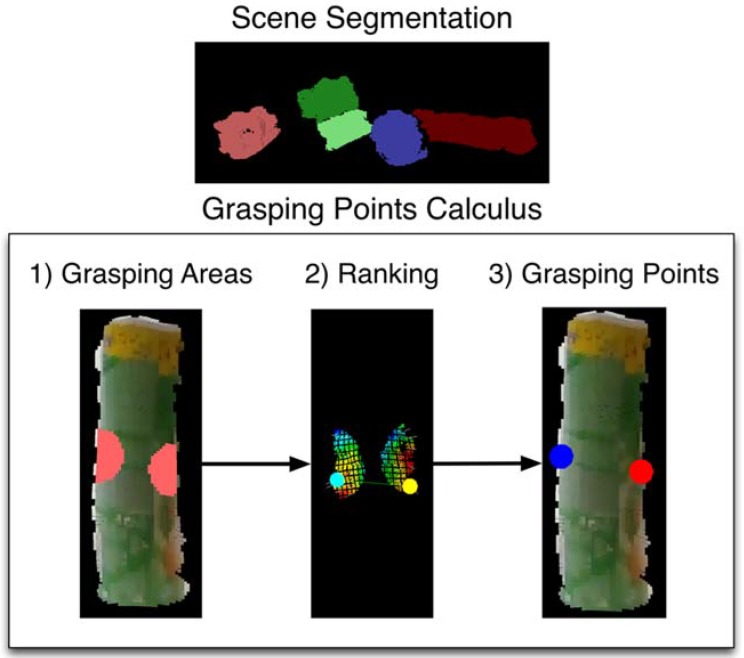
Steps of the method for calculating a pair of contact points. Scene Segmentation: clouds of the detected objects. Grasping Points Calculus, executed for each detected object: (1) grasping areas with potential contact points, (2) curvature values and a pair of evaluated contact points, (3) best ranked pair of contact points.

The proposed method consists of two phases. First, the vision algorithm detects the presence of unknown objects on the scene, segments the scenes to obtain clusters of each object (each cluster is a point cloud) and then, it computes grasping points on the surface of each of the objects (Figure 4). The method is flexible to obtain grasping points of objects even changing the scenario providing that objects are located on a table or flat surface. Once the vision algorithm provides the robot with the optimal grasping points of the object, the robot plans the trajectory in order to position the robot hand to grasp the object. Occasionally, the grasping of the object is not optimal. For this reason, the method adds a second phase which is used to plan fine hand robot-object interactions. In this step, EMG-based teleoperation of the robot hand-arm is performed to accomplish a successful and stable grasp without slipping and avoiding damage to the object.

### 3.1. Grasping Points and Pose Estimation

The algorithm calculates pairs of contact points for unknown objects given a single point cloud captured from a RGBD sensor with eye-to-hand configuration. Firstly, the point cloud is segmented in order to detect the objects present in the scene. Then, for each detected object, the algorithm evaluates pairs of contact points that fulfil a set of geometric conditions. Basically, it approximates the main axis of the object using the major vector obtained by running a Principal Component Analysis (PCA) extraction. Then, it calculates the centroid in the point cloud. With this information, it is possible to find a cutting plane perpendicular to the main axis of the object through its centroid. The candidate contact areas are at the opposite edges of the surface of the object that are close to the cutting plane. A standard grasping configuration consists of one point from each of these two areas. Figure 4 shows all these steps graphically.

These candidate areas, in which the robot hand can be positioned, contain multiple potential points so the vision algorithm evaluates a great variety of grasping configurations for the robot hand, using a custom metric that ranks their feasibility. Thereby, the best-ranked pair of contact points is selected, since it is likely to be the most stable grasp, given the view conditions and the used robotic hand. The algorithm takes into account four aspects: the distance of the contact points to the cutting plane, the geometric curvature at the contact points, the antipodal configurations and the perpendicularity to the contact points.

The first one, distance of the contact points to the cutting plane, is important because it is assumed that the grasping of the object is more stable as the robotic hand grasps closer to the centroid of the object, which is an approximation of its centre of mass. This way, the inertial movements caused throughout the manipulation process of the object are more controllable. The second aspect, the curvature, is considered to avoid the grasps of unstable parts on the object surface. The goal is to place the fingertips on planar surfaces instead of highly curved areas that are prone to be more unstable. Grasping objects on non-planar areas can cause a slip and fall of a grasped object when it is being manipulated, for example, if the robot arm executes a lifting movement. Regarding to the third aspect, contact points should be located on places where the robotic fingers can apply opposite and collinear forces (antipodal configuration). Finally, it is desirable to have contact points that are connected by a line perpendicular to the main axis of the object. That is, the contact points are equally distanced from the cutting plane.

The aforementioned aspects are used to define a quality metric to evaluate the candidate contact point and to propose the best grasp points to carry out a successful grasp of the object on the scene. Accordingly, this quality metric ranks with greater values the grasping configurations that place the robotic hand with its palm point towards the object, its fingertips perpendicular to the axis of the object, parallel to the cutting plane and close to the centroid of the object. Notice that this operation is performed for every detected object. Consequently, the final pose of the robot hand is calculated using the best ranked grasping configuration and the approximated main axis of the object.

Our vision algorithm only computes pairs of contact points. This is assumed to avoid the method being dependent on the type of robotic hand mounted at the end of the robotic arm. Two points are the minimum required for a simple robotic gripper but also, any multi-finger robotic hand can adapt its grasping configuration to two points on the object surface. In the experiments, we use an Allegro hand with four fingers, one of which acts as the thumb. In practice, it is assumed that the grasps will be done with three fingers. This number has been limited to three because the Allegro hand size is often bigger than the object size which will be grasped.

In order to perform three-finger grasps, the algorithm takes into account the following criterion: one of the contact points corresponds to the place the thumb must reach during a grasp, while the other contact point remains between the first two fingers (index and middle). This means that the first and second finger wrap around the second contact point. In this way, the grasp adapts its configuration to only two contact points even though the hand uses three fingers. In addition, the robotic hand is oriented perpendicular to the axis of the object, meaning that it adapts to the pose of the object.

When the human operator has selected the desired object that will be grasped, the robotic system guided by the vision algorithm performs the following steps to reach it:

1. First, the robotic hand is moved to a point 10 cm away from the object. This is a pre-grasping position which is used to facilitate the planning of the following steps. The pre-grasping position is computed, from location (position and orientation) of contact points on the object surface, by the vision algorithm previously described.
2. Second, the robotic hand is moved forward facing the object with its palm and the fingers opened. In this step the hand reaches the point in which, after closing, it would place the fingertips on the calculated contact points.

The correctness of this position depends on the calibration of the camera position with regards to the world’s origin as well as lighting conditions and reflectance properties of the objects in the scene. Owing to this, the proposed method performs the correction of the robot hand using the sEMG signals. But also, sEMG can be used to accomplish a proper grasp of objects in a complex manipulation.

### 3.2. Collaborative System with Both Visual and Electromyography Data

The proposed solution has been implemented using the ROS in order to develop nodes in charge of different responsibilities but keeping a communication framework among them. One node has been created, called *pointcloud_listener*, where point clouds are read and processed to perform the calculus of the grasp contacts. This node publishes a custom ROS message called *GraspConfiguration* where the point clouds of the objects and the calculated grasp contacts are stored.

Another node, called *allegro_control_grasp*, subscribes to this topic and reads the published contact points to generate a grasp pose for the robotic gripper. Then, it proceeds to plan a trajectory following the steps listed in the previous section. MoveIt! [29] has been used to perform this trajectory planning. Once it reaches the grasping position, the EMG control starts. To do so, it subscribes to a topic called/*emgsensor*/*move* where the correcting movements are published.

These corrections are published by a third node called *emg_reader*, which processes the sEMG signals in order to provide messages of type *geometry_msgs*/*Quaternion*. This type of ROS message allows us to describe the direction of movement for the arm that the operator wants to perform in order to correct the position of the robotic gripper. Thus, using one of the axis of the Quaternion, we can specify in which axis we want to move the gripper. The *w* term is set to 1 when we detect the grasping pattern in the EMG signal so the *allegro_control_grasp* node closes the gripper and continues to lift and carry the object.

**Figure 5:**
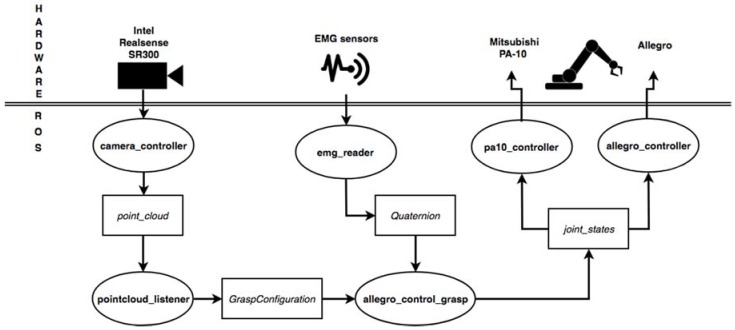
Scheme of the proposed method implemented in Robot Operating System (ROS) showing communication modules among different steps.

It is important to note that this message is constantly published by the *emg_reader* node but the *allegro_control_grasp* only reads them after performing a correction. This means that messages published during the physical movement of the robot are ignored and, as soon as it stops, the control returns to wait for a new message in the topic. Figure 5 shows a scheme of the nodes and their interactions through ROS.

## 4. Experiments and Discussion

### 4.1. Test Design

Six subjects (age 24.5 ± 6.2 years old, four male and two female) without previous experience on myoelectric control participated in the experimental tests. First, subjects were asked to perform several wrist flexion and extensions at different force levels and thresholds were visually chosen from the processed sEMG signals of the FDS and ECR. After selecting the proper thresholds, subjects were asked to freely perform wrist contractions and the classification output was shown to them until they felt comfortable with the myoelectric setup.

The experimental tests were divided into three sets of grasping activities, each one for a different positioning of the object. The object, a cylindrical plastic can (23 cm height, 8 cm diameter), was placed vertically (position 1), horizontally (position 2) and in a diagonal orientation (position 3). Each grasping activity was performed five times for each position and subject. Subject 5 did not perform the last set (position 3) of grasping tasks due to fatigue and technical problems.

During the grasping activity, the visual-driven robot arm positioned the robotic hand facing the side of the object and then, subjects were asked to readjust the z-axis (weak wrist extension or flexion) and then grasp the object voluntarily with a strong wrist flexion. The accuracy of classifying sEMG signals was measured by counting correct sEMG commands (classification success), no detections (if muscle contraction was present but the control command was not generated) and errors in the classification output. No detections were manually counted from the visualization of correct contractions that did not reach the selected thresholds. Errors were counted as wrong generated commands. Grasping accuracy was measured by counting correct graspings of the object, i.e., if the object did not flip or fall from the robotic hand.

### 4.2. Results and Evaluation

Table 1, Table 2 and Table 3 show the results obtained on sEMG performance (classification success, no detection, classification error) and grasping performance in terms of accuracy (ACC), i.e., percentage of correct grasps. sEMG accuracy was obtained by dividing successful classifications by performed contractions.

From the results, it can be concluded that both sEMG and grasping accuracy is high. sEMG errors or no detections do not always affect grasping accuracy as the robot hand is quite well positioned with the visual-driven system alone. It is interesting to notice that for object position 2 the grasping is always successful. This is possibly due to the fact that the object is placed horizontally to the ground and, as it is cylindrical, it sometimes rolls until touching the thumb of the hand when the hand is repositioned. Nevertheless, grasping for the remaining object positions is also very accurate (93.33% ± 10.33% for position 1 and 92.00% ± 10.95% for position 3). Regarding sEMG classifications, errors are fewer than no detections. A possible solution to reduce these errors is a longer training of the subjects (in these tests, subjects were naïve to myoelectric control systems). Another option could be the use of a more conservative threshold selection. This will prevent the appearance of errors but would probably increase the no detections increasing the time taken to perform the grasping.

The results of a previous experiment, in which only the visual-driven system was used, are compared, in Table 4, to the results of the proposed sEMG-based system. Visual-driven tests are automatic, so there is no direct implication of a human operator in the positioning of the robot and the following grasping. The error for experiments without EMG represents two kind of errors. One of them is due to the slipping of the object during the grasping tasks. Other errors occurred because the hand position is not properly fit with vision techniques. Both cases are mostly solved when sEMG control is added to the grasping system. This way, sEMG can be used to correct the hand pose and its grasps, showing an increase in grasping accuracy close to a 9% using the same cylindrical object. Besides, the accuracy increases up to a 15% if it is compared with other grasping experiments using other cylindrical objects Consequently, the average increase in accuracy is around 13.8% considering the 81 trials without sEMG.

## 5. Conclusions

In this paper, we propose a method based on combining both computer vision and sEMG techniques to allow a human operator to carry out grasping tasks of objects. The proposed method has been demonstrated and validated by several human operators with different ages and sex. To do this, our method uses a vision algorithm to estimate grasping points on the surface of the detected object and moves the robotic hand-arm system from any pose to a pre-grasping pose according to the object. Then, sEMG signals from arm muscles of human operators are measured, processed and transformed into movements of the robotic hand-arm system. Thereby, the human operator can readjust the robotic hand to properly grasp the object. The results show an increase of around a 9% in grasping accuracy compared to the use of the visual-driven system alone with the same object and around a 15% with similar cylindrical objects.

The proposed method evaluates a simple ON/OFF myocontrol classification algorithm based on a threshold selection with a very high reliability and that could be easily translated into an industrial environment with the introduction of low-cost sEMG devices such as the MYO Thalmic bracelet or Arduino-based acquisition systems. Additionally, specific expertise is not needed to instrument the sEMG system, as the location of electrodes on flexor and extensor muscles is straight-forward. This is a first approach towards bridging the gap between human operators with and without disabilities in industrial works in which grasping and manipulation tasks are required. In the future, we hope to integrate more signals to control additional degrees of freedom during the movement to generate better grasps and more complex manipulation tasks.

## Figures and Tables

**Table 1:** sEMG performance and grasping accuracy for object position 1.

Subject	Success	Error	No Detection	sEMG ACC	Grasping ACC
**A01**	10	0	0	100%	100%
**A02**	10	0	1	91%	100%
**A03**	10	1	2	77%	100%
**A04**	8	1	0	89%	100%
**A05**	10	0	0	100%	80%
**A06**	6	2	1	67%	80%
**Average**	9.00	0.67	0.67	87.23%	93.33%
**Standard deviation**	1.67	0.82	0.82	13.20%	10.33%

**Table 2:** sEMG performance and grasping accuracy for object position 2.

Subject	Success	Error	No Detection	sEMG ACC	Grasping ACC
**A01**	8	1	0	89%	100%
**A02**	10	1	1	83%	100%
**A03**	10	0	1	91%	100%
**A04**	8	1	0	89%	100%
**A05**	10	1	3	71%	100%
**A06**	10	0	2	83%	100%
**Average**	9.33	0.67	1.17	84.46%	100.00%
**Standard deviation**	1.03	0.52	1.17	7.12%	0.00%

**Table 3:** sEMG performance and grasping accuracy for object position 3.

Subject	Success	Error	No Detection	sEMG ACC	Grasping ACC
**A01**	10	0	1	91%	80%
**A02**	10	0	0	100%	100%
**A03**	10	1	0	91%	100%
**A04**	10	0	1	91%	100%
**A06**	8	1	0	89%	80%
**Average**	9.60	0.40	0.40	92.32%	92.00%
**Standard deviation**	0.89	0.55	0.55	4.38%	10.95%

**Table 4:** Comparison of the grasping accuracy for the proposed (visual data + sEMG) compared to the previous method (only visual data).

Subject	Trials	Success	Error	Grasping ACC
with sEMG	85	81	4	95.29%
without sEMG (same object)	15	13	2	86.66%
without sEMG (other cylindrical objects)	66	53	13	80.30%
